# BRICKS FOR BETTER BRAINS©: STRATEGIES TO ENHANCE ENGAGEMENT IN PERSONS LIVING WITH DEMENTIA

**DOI:** 10.1093/geroni/igae098.3813

**Published:** 2024-12-31

**Authors:** Natalia Kasperovich, Jennifer Sasser

**Affiliations:** Bricks for Better Brains©, Beaverton, Oregon, United States; Portland Community College, PORTLAND, Oregon, United States

## Abstract

The Bricks for Better Brains© activity is an intervention to slow down the progression of cognitive decline for persons living with dementia. The activity utilizes various carefully selected, interlocking bricks (LEGO®) in bright colors as an innovative medium for stimulating participants’ creative capacities, using visual perception and motor sensory skills, as well as strengthening social engagement. Cognitive impairments can present unique challenges for effectively engaging this population in meaningful activities. An in-person observational study was conducted as a preliminary exploration of strategies to increase engagement of persons living with dementia in activities with viable therapeutic potential using The Bricks for Better Brains© protocol. The study consisted of two groups (five males and fifteen females total) of people 75 years of age and older living with moderate-to-severe dementia and residing in an institutional care setting. Four activity modification strategies were tested – modification to objects and property; modification to space demands; modification to social demands; and modification to sequence and timing. All modifications in space demands increased engagement. Modification in objects and property, sequence and timing, and social demands were positive for the group but showed mixed efficacy for individual. These results support the person-centered care model and highlighted the importance of creating stimulating environments by understanding how people living with dementia interact with their surroundings. By identifying efficient activity modification strategies we can increase activity engagement and improve the quality of life of the people living with dementia. Future studies will need to include the reliability of observational measures.

